# Effect of peep on esophageal catheter optimal calibration volume and esophageal pressure measurements

**DOI:** 10.1186/2197-425X-3-S1-A1001

**Published:** 2015-10-01

**Authors:** F Magni, D Ceriani, L Castagna, C Ferrari, A Zanella, V Scaravilli, SM Colombo, M Laratta, N Patroniti

**Affiliations:** Università degli Studi di Milano Bicocca, Monza, Italy; San Gerardo Hospital, Cardiac Intensive Care Unit, Monza, Italy; San Gerardo Hospital, Intensive Care Unit, Monza, Italy

## Introduction

The use of esophageal balloon catheter to estimate pleural pressure has gained renewed popularity in recent years. Indeed, measurement of transpulmonary pressure may allow a more pathophysiological-based approach to ventilator strategy in ARDS patients. Nevertheless it is well known that esophageal balloon catheter derived parameters can be influenced by several patient-related or technical-related factors.

## Objectives

To evaluate in-vivo the effect of positive end-expiratory pressure (PEEP) variations on esophageal catheter optimal calibration volume and measured esophageal pressure.

## Methods

Experimental study in 8 (5 ARDS, 3 control) sedated, intubated, paralyzed and mechanically ventilated (volume-control) patients. Patients were monitored with esophageal balloon catheter (*Cooper Surgical, Trumbull, CT USA*).

Three PEEP groups were defined: low_PEEP_ (8 and 4 cmH_2_O respectively in ARDS and control patients), medium_PEEP_ (12 and 8 cmH_2_O) and high_PEEP_ (16 and 12 cmH_2_O).

During each PEEP level, we inflated the esophageal balloon with increasing amount of air (from 0.2 to 2 ml). For each injected volume, we performed an end-inspiratory occlusion maneuver followed by an occlusion test by applying manual chest compression during an end-expiratory airway occlusion maneuver. We measured the ratio between airway pressure variation and esophageal pressure variation (ΔPaw/ΔPes ratio) during the occlusion test, end-expiratory esophageal pressure (Pes,e), end-expiratory transpulmonary pressure (Pl,e), chest wall compliance (Cpl_CW_), lung compliance (Cpl_L_), elastance-derived transpulmonary plateau pressure (ΔPl,i). The optimal calibration volume (defined as the injected volume at which ΔPaw/ΔPes ratio was closer to 1) was identified for each PEEP group (VC_LPEEP_ for low_PEEP_, VC_MPEEP_ for medium_PEEP_, VC_HPEEP_ for high_PEEP_). Effect of PEEP on derived parameters was assessed by comparing at PEEP medium and high values obtained at the VC_LPEEP_ against values obtained with the optimal VC at each PEEP.

## Results

**Figure 1:**
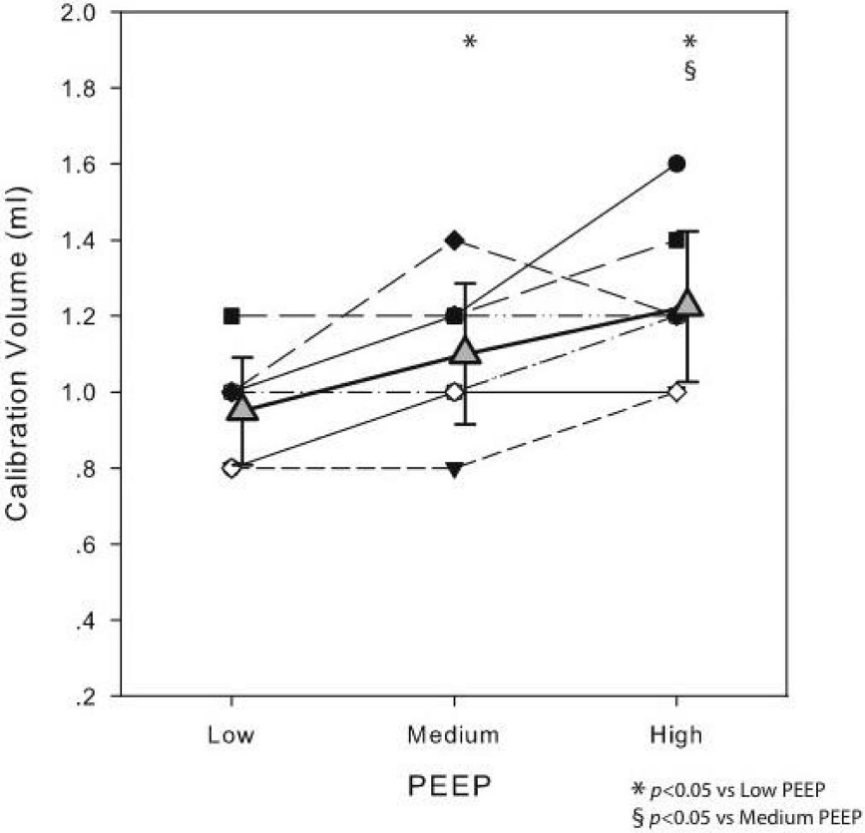
**Optimal calibration volume and PEEP**

Optimal calibration volumes progressively raised with increasing PEEP (0.95 ± 0.14 ml, 1.1 ± 0,18 ml, 1.22 ± 0.2 ml respectively for low_PEEP_, medium_PEEP_ and high_PEEP_; *p*< 0.001). See Figure 1. At high PEEP, Pes,e, Cpl_L_ and ΔPl,i were significantly higher while Cpl_cw_ was significantly lower e when measured with VC_HPEEP_ compared to VC_LPEEP_.

Partitioned respiratory system mechanic parameters are show in Table.

## Conclusions

Esophageal catheter balloon calibration volume is affected by PEEP. Neglecting this effect may leads to errors in computing partitioned respiratory system mechanics. Catheter calibration should be checked after every change in PEEP.

**Figure:**
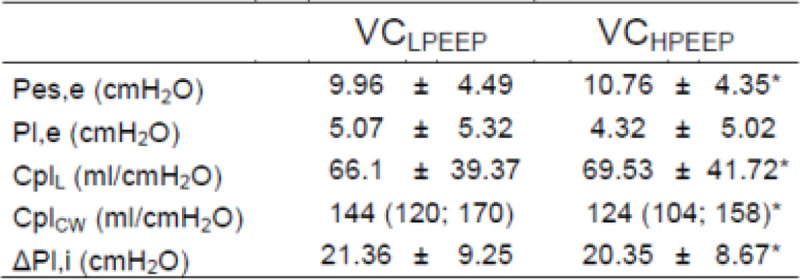
Figure 2
